# Integrative analysis and experiments to explore angiogenesis regulators correlated with poor prognosis, immune infiltration and cancer progression in lung adenocarcinoma

**DOI:** 10.1186/s12967-021-03031-w

**Published:** 2021-08-21

**Authors:** Songhua Cai, Xiaotong Guo, Chujian Huang, Youjun Deng, Longde Du, Wenyi Liu, Chenglin Yang, Hongbo Zhao, Kai Ma, Lixu Wang, Jie He, Zhentao Yu

**Affiliations:** 1 Department of Thoracic Surgery, National Cancer Center/National Clinical Research Center for Cancer/Cancer Hospital & Shenzhen Hospital, Chinese Academy of Medical Sciences and Peking Union Medical College, Shenzhen, 518116 China; 2 Department of Thoracic Surgery, National Cancer Center/National Clinical Research Center for Cancer/Cancer Hospital, Chinese Academy of Medical Sciences and Peking Union Medical College, Beijing, 100021 China

**Keywords:** Angiogenesis, LUAD, Immune checkpoint, Chemotherapy

## Abstract

Angiogenesis is the process of capillary sprouting from pre-existing vessels and it plays a critical role in the carcinogenic process of lung adenocarcinoma (LUAD). However, the association of angiogenesis regulators with the prognosis and progression of LUAD needs to be further elucidated. In this study, we adopted differential expression analysis, Cox proportional hazards (PH) regression analysis and experimental validation to identify angiogenesis regulators correlated with a poor prognosis, immune infiltration and cancer progression in LUAD. These results showed that the diagnostic and prognostic models based on COL5A2 and EPHB2 served as independent biomarkers with superior predictive ability. The patients in the high-risk group exhibited a worse prognosis in the TCGA cohort (P < 0.001, HR = 1.72, 95% CI 1.28–2.30), GSE310210 cohort (P = 0.005, HR = 2.87, 95% CI 1.46–5.61), and GSE31019 cohort (P = 0.01, HR = 2.14, 95% CI 1.19–3.86) than patients in the low-risk group. The high prognostic risk patients had a higher TMB (P < 0.001); higher fractions of M0 macrophages, neutrophils, NK cells resting, and T cells CD4 memory activated (P < 0.05); and higher expression of immune checkpoints PD-1, PDL-1, PDL-2, and B7H3 (P < 0.001). Patients in the high-risk group were more sensitive to chemotherapeutic drugs and molecular targeted drugs such as cisplatin, doxorubicin, gefitinib, and bosutinib (P < 0.0001). In addition, inhibition of COL5A2 and EPHB2 effectively suppressed the proliferation and migration of LUAD cells. The current study identified angiogenesis regulators as potential biomarkers and therapeutic targets for LUAD and may help to further optimize cancer therapy.

**Supplementary Information:**

The online version contains supplementary material available at 10.1186/s12967-021-03031-w.

## Background

Lung cancer is the most common leading cause of cancer-associated death (18.4%) and the most frequent malignant tumor (11.6%) worldwide [1]. Lung adenocarcinoma (LUAD) is the most common histological type of lung cancer, accounting for approximately 40% of all lung cancer cases [2]. Once LUAD is diagnosed, conventional methods, including surgery, chemotherapy, immunotherapy, radiotherapy, and targeted gene therapy, are used to treat the patients [3]. Although cancer-related treatment technology has gradually improved, the five-year survival rate is still poor, which is partly because LUAD is often diagnosed as advanced or metastatic disease [4]. The underlying molecular mechanism and promising biomarkers of LUAD are complex, which needs to be further elucidated.

In recent years, high-throughput sequencing technologies (such as microarray and RNA-seq) combined with experiments have become effective methods to explore the value of biomarkers for the early diagnosis and prognostication of patients with malignancies, including liver cancer and LUAD [5, 6]. Based on multiomics analysis, Huang et al. identified AURKB as a potential prognostic biomarker and therapeutic target for lung adenocarcinoma [7]. Zhang et al. proved that AURKA and FAM83A were novel targets to improve the prognosis of patients with LUAD and were correlated with immune infiltration [8]. Due to the limited reliability of a one-gene prognostic model, some researchers constructed multigene signature models. For example, Wang et al. identified four prognostic genes (CD69, KLRB1, PLCB2, and P2RY13) related to immune infiltration in lung adenocarcinoma through univariate prognostic analysis and Lasso-Cox regression [9]. The predictive ability of these signature models was higher than that of the one-gene prognostic models. It is also beneficial to explore diagnostic and prognostic biomarkers while considering potential molecular mechanisms of LUAD, such as DNA repair, apoptosis, and angiogenesis.

Angiogenesis is the process of new blood vessel formation from pre-existing vessels, which is regarded as a hallmark of malignant tumors, and it plays a central role in cancer occurrence, development and metastatic dissemination [10, 11]. Targeting angiogenesis in LUAD is considered an effective treatment strategy, and exploring efficient molecular diagnostic and prognostic markers based on the regulatory mechanisms of angiogenesis regulators may help further improve the clinical outcomes and individualized treatment strategies for patients with LUAD [12]. In a previous study, tumor angiogenesis was proven to have a positive correlation with many solid tumors, such as lung cancer, colorectal cancer, gastric cancer, and breast cancer [13, 14]. Important predictive diagnostic and prognostic biomarkers of angiogenesis-related genes have been identified in some malignancies, including liver cancer, colorectal cancer, gastric cancer and urothelial cancer [15–17]. Thus, the correlation of the angiogenesis gene signature and clinical outcomes or clinical relevance needs to be elucidated in LUAD patients.

The tumor microenvironment (TME) is a complex network that comprises various cell types, cytokines and other extracellular components and plays a fundamental role in cancer recognition and treatment [18]. Over the past decade, immunotherapy targeting specific tumor-infiltrating immune cells (TIICs) and immune checkpoint inhibitors (ICIS) has been proven to improve the therapy response and clinical outcome of patients with LUAD [19]. Tumor-infiltrating immune cells, which are effectors and responders of the immune system, are the key to our understanding of tumor cell escape from human immune surveillance [20]. Tumor angiogenesis has been shown to be closely associated with immune infiltration, and proangiogenic regulators may be involved in immunotherapy and cancer progression [21]. Thus, it is urgent to explore the promising and effective angiogenesis molecular prognostic markers in LUAD patients.

In this study, we analyzed 154 angiogenesis-related genes in the LUAD cohort by using TCGA and GEO databases. Based on univariate Cox proportional hazards (PH) regression and multivariate Cox PH regression analyses, we constructed two angiogenesis-related gene (COL5A2 and EPHB2) models for predicting the diagnosis and prognosis of LUAD. Then, we explored the relationship between the angiogenesis signature and immune cell infiltration in LUAD patients. In addition, we knocked down the levels of COL5A2 and EPHB2 in LUAD cells and measured the anti-proliferative and anti-migration effects. These findings may help further improve the early diagnosis rate and the development of personalized treatment strategies for LUAD patients (see Additional File 1: Figure S1).

## Materials and methods

### Identification of differentially expressed genes (DEGs) and functional enrichment analysis between LUAD and nontumor tissues

The mRNA-sequencing data of LUAD patients with clinical information were downloaded from the TCGA database (http://www.tcga.org/) (including 59 non tumor samples and 535 LUAD samples). Angiogenesis-related genes were obtained from previous research (Additional File2: Table S1). The differentially expressed genes (DEGs) involved in angiogenesis in the LUAD patients were identified with the “limma” R package (absolute log2-fold change (FC) > 1 and an adjusted P value < 0.05), in which we used the Benjamini-Hochberg (BH) method to obtain false discovery rate (FDR) as the adjusted P value. The DEGs were collected to perform functional enrichment analysis. The GO functional enrichment analysis and KEGG functional enrichment analysis were performed in the Metascape website (https://metascape.org/).

### Establishment and validation of angiogenesis-related prognostic model in patients with LUAD

Univariate Cox PH regression and multivariate Cox PH regression analyses were used to identify the genes that were significantly associated with the OS of LUAD patients (P < 0.05). And the PH assumption for the COX model was validated using the “coxph” function in the survival package in R software. The prognostic index (PI) = (β* expression level of COL5A2) + (β* expression level of EPHB2). A medium PI was regarded as the optimal cutoff, and LUAD patients were divided into two groups: a high-risk group and a low-risk group. Kaplan–Meier (K–M) curves and time-dependent ROC curves were used to validate the predictive performance of the angiogenic gene prognostic model.

### Construction and evaluation of a predictive nomogram

Univariate and multivariate Cox PH regression analyses were performed to assess the independent prognostic factors (including age, sex, T stage, N stage, M stage, TNM stage and risk score of angiogenesis-related gene signature) of LUAD patients. Then, all of the independent prognostic factors were used to establish a nomogram to predict the survival probability of LUAD patients at 1 year, 3 years and 5 years. The calibration curve and ROC curve were used to evaluate the predictive performance of the nomogram.

### Gene set enrichment analysis

Gene set enrichment analysis (GSEA) was adopted to identify the signaling pathway of the angiogenesis-related gene signature in LUAD patients. P < 0.05 was considered statistically significant. The pathways used for GSEA were obtained from the Molecular Signatures Database (MSigDB) (http://software.broadinstitute.org/gsea/msigdb).

### Estimation of immune cell type fractions

The 22 immune cell type fractions of patients with LUAD were estimated by the “CIBERSORT” R package. For each tissue, the total fractions of 22 immune cell types were equal to 1. The estimated immune cell type scores of patients with LUAD were compared between the high-risk group and the low-risk group.

### Cell culture and siRNA administration

The human LUAD cell lines, including A549 cells and H1299 cells, were purchased from the American Type Culture Collection (ATCC) (Manassas, VA, USA). The A549 and H1299 cells were cultured in DMEM (Gibco, NYC, USA) containing 10% fetal bovine serum (FBS) (Gibco, NYC, USA) and 100 U/ml penicillin–streptomycin (Gibco, NYC, USA) in an incubator with 5% CO_2_ at 37 °C. The cells were transfected with 50 nM COL5A2 siRNA or EPHB2 siRNA in basic DMEM for 6–8 h and then changed to DMEM supplemented with 10% FBS and 100 U/ml penicillin–streptomycin. The A549 and H1299 cells were treated with COL5A2 siRNA or EPHB2 siRNA for 48 h for the next experiment.

### Western blot assay

SDS-PAGE Sample Loading Buffer (Beyotime, SHH, CHN) was used to collect the total protein from the A549 and H1299 cells. Protein samples were separated using 8–10% sodium lauryl sulfate–polyacrylamide gel electrophoresis (SDS–PAGE) and then transferred to a polyvinylidene fluoride (PVDF) membrane before blocking at room temperature for 1 h in 5% skim milk. Next, the membrane was incubated with the primary antibody at 4 ℃ overnight and then incubated with the secondary antibody at room temperature. The image was measured by an iBright FL1000 intelligent imaging system (Invitrogen, Carlsbad, USA).

### Cell proliferation assay

The proliferation of the A549 and H1299 cells was measured by a Cell Counting Kit-8 (CCK8) assay (Beyotime, SHH, CHN) and 5-ethynyl-2′-deoxyuridine (EdU) kit (Beyotime, SHH, CHN). After COL5A2 siRNA or EPHB2 siRNA treatment, A549 and H1299 cells were seeded in 96-well plates with 5000 cells and 200 µL DMEM per well for 48 h and then incubated with the EdU kit for 3 h following the manufacturer’s protocol. Alternatively, the cells were cultured for 0–72 h and incubated with the CCK-8 kit following the manufacturer’s instructions for 2 h. A microplate reader (Bio-Rad, Berkeley, CA, USA) was used to detect the absorbance of the cells at 450 nm.

### Cell migration assay

Transwell assays were performed to detect the migration of the A549 and H1299 cells. After COL5A2 siRNA or EPHB2 siRNA treatment, the A549 and H1299 cells were cultured in the upper chambers of 24-well culture plates with 5 × 10^4^ cells and 200 µl serum-free DMEM. The lower chambers of these 24-well culture plates were incubated with DMEM supplemented with FBS and penicillin–streptomycin. The cells were cultured in an incubator with 5% CO_2_ at 37 °C. Then, cells on the lower membrane were stained with 0.1% crystal violet, and the migrated cells were counted under a light microscope.

### Statistical analysis

R studio (version 4.0.3) and GraphPad Prism software (version 6.0) were used to compare the differences between the experimental and control groups. COX PH regression and K-M non-parametric analysis for the prognostic model were performed using the “survival” and “survminer” packages in R software.The experimental results are shown as the mean ± standard deviation (SD) of at least three independent experiments. A P value < 0.05 was adopted to calculate statistically significant differences.

## Results

### Differentially expressed angiogenesis-related genes and functional enrichment analysis of LUAD patients

**Fig. 1:**
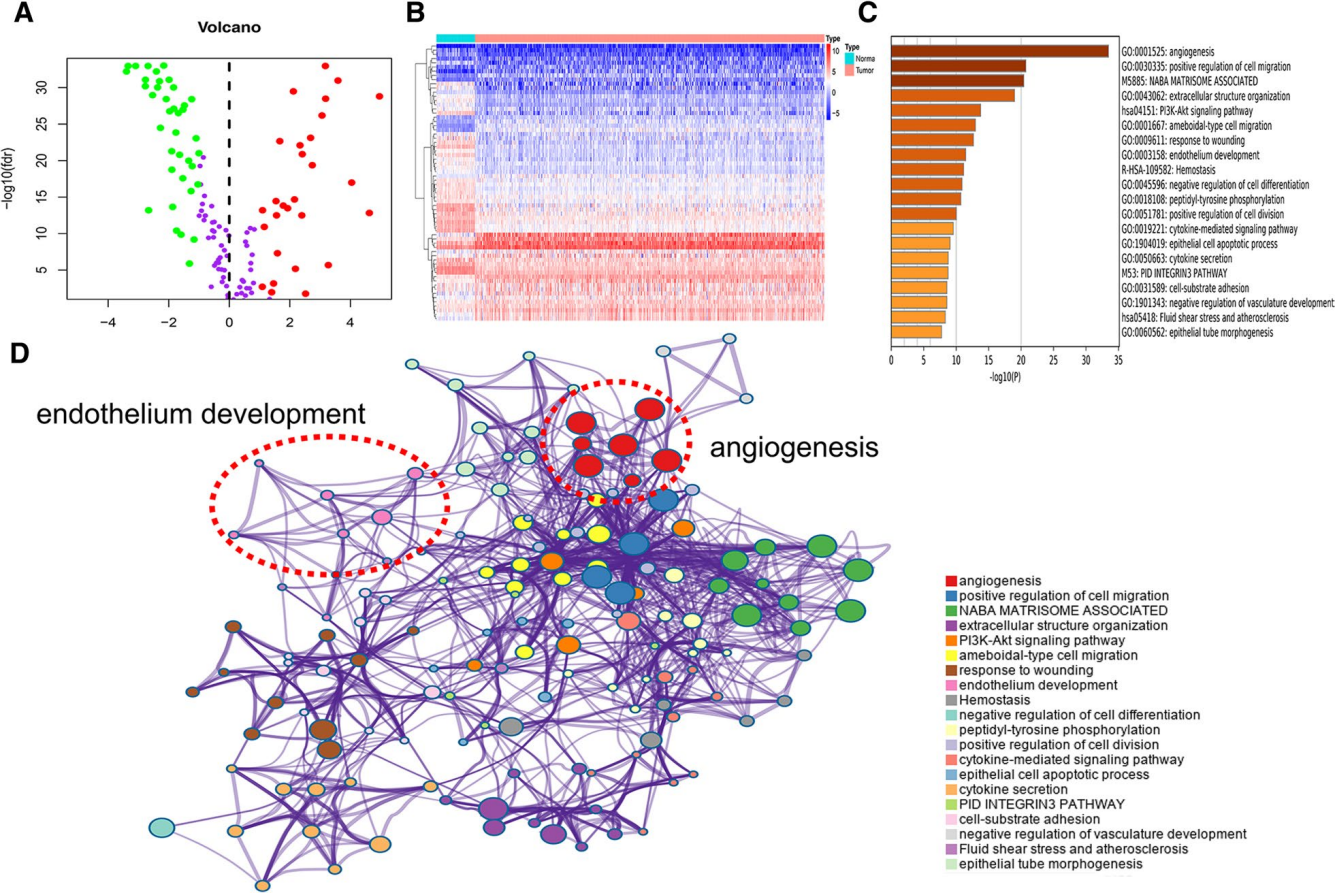
Identification of angiogenesis-related DEGs and functional enrichment analysis in LUAD patients. **A**, **B** The heatmap and volcano plot indicate the differentially expressed angiogenesis-related genes of LUAD from the TCGA database. **C**,** D** GO functional enrichment analysis and KEGG pathway analysis of 66 DEGs by the Metascape website

A total of 154 angiogenesis regulators were obtained from previous research on angiogenesis and then matched to the mRNA sequence of these genes in the TCGA-LUAD cohort. Then, we identified differentially expressed genes (DEGs) using the Limma R package (absolute log2-fold change (FC) > 1 and an adjusted P value < 0.05) between LUAD samples and adjacent noncancerous samples. We identified 66 differentially expressed angiogenic genes (28 upregulated and 38 downregulated) in the TCGA-LUAD cohort (Fig. 1A, B). We performed KEGG pathway analysis and GO functional enrichment analysis of these genes through the Metascape website (https://metascape.org/). The top 20 genes were enriched in signaling pathways such as angiogenesis, endothelial development, the PI3K-Akt signaling pathway, and cytokine secretion (Fig. 1C, D).

To analyze these 66 differentially expressed angiogenic genes that are associated with LUAD survival, we used univariate Cox PH regression analysis to explore the prognostic role of these genes among LUAD patients (Additional File 3: Table S2). Then, we performed multivariate Cox PH regression analysis to further identify angiogenic genes (COL5A2 and EPHB2) that were significantly associated with the OS of LUAD patients (Additional File 3: Table S2).

### Measuring the oncogenic effect of COL5A2 and EPHB2 in LUAD cells

**Fig. 2:**
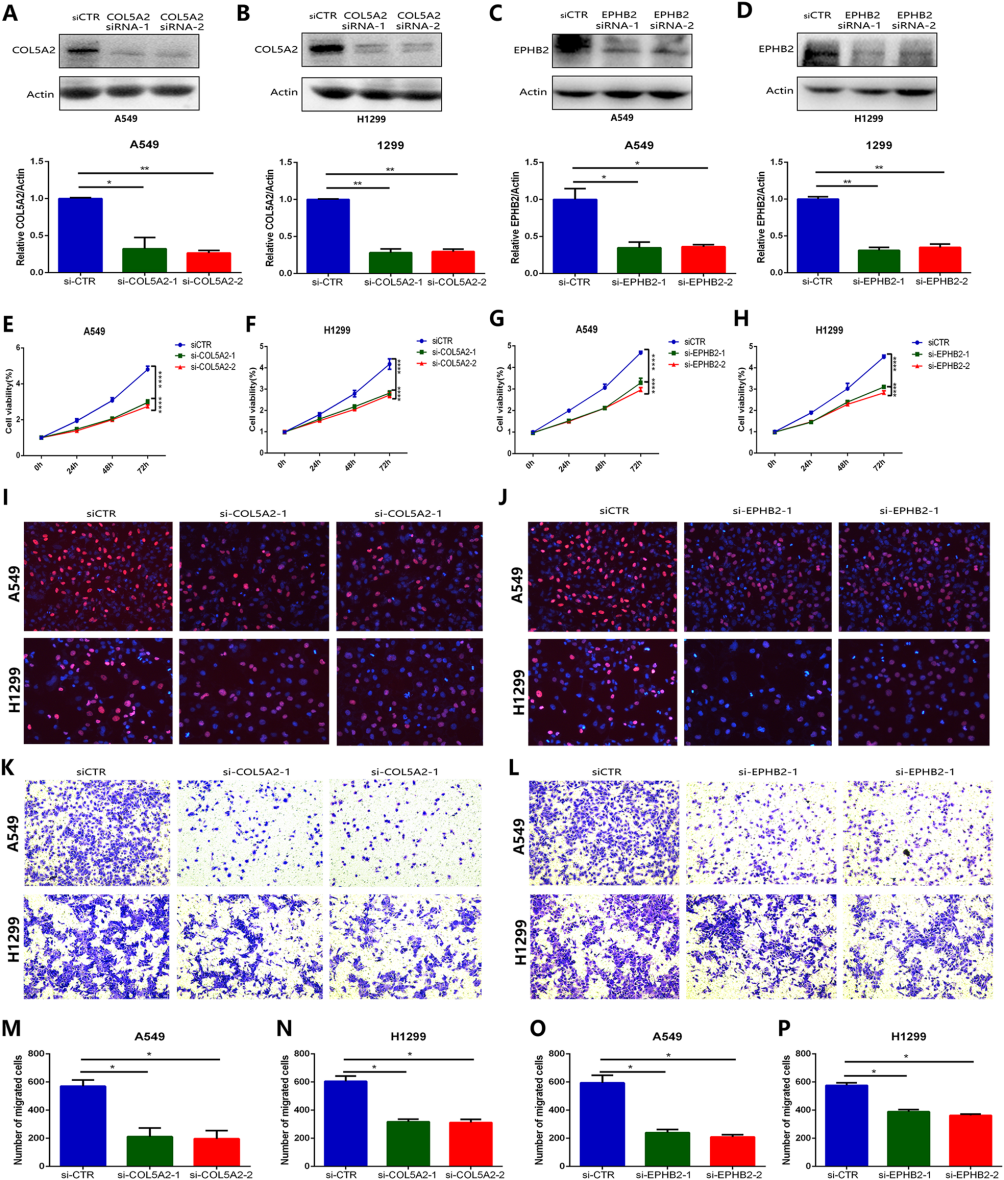
The oncogenic effect of COL5A2 and EPHB2 in LUAD cells. **A**–**D** Western blot analysis indicated the inhibitory efficiency of COL5A2 siRNA and EPHB2 siRNA in A549 cells and H1299 cells. **E**–**H** CCK-8 assay showed the proliferation of A549 cells and H1299 cells treated with COL5A2 siRNA and EPHB2 siRNA. **I**, **J** EdU assay revealed the proliferation of A549 cells and H1299 cells with COL5A2 inhibition and EPHB2 inhibition. **K**, **L** Transwell assays revealed the migration of A549 cells and H1299 cells treated with COL5A2 siRNA and EPHB2 siRNA. **M**–**P** Quantitative statistical results of the number of effects of migration of A549 cells and H1299 cells. Data are shown as the mean ± SD of at least three independent experiments. *P < 0.05, **P < 0.01, ***P < 0.001

To explore the role of COL5A2 and EPHB2 in LUAD cell proliferation and migration, we used siRNA to knock down the expression levels of COL5A2 and EPHB2 in LUAD cells (including A549 and H1299 cells). Western blot analysis revealed that the levels of COL5A2 and EPHB2 were significantly inhibited by the siRNAs of these two genes (Fig. 2A–D). Then, CCK-8 assays (Fig. 2E–H) and EdU assays (Fig. 2I, J) were used to measure LUAD cell proliferation. The proliferation of the A549 and H1299 cells was effectively suppressed with COL5A2 and EPHB2 inhibition (Fig. 2E–J). In addition, a Transwell assay was performed to detect migration of the LUAD cells. The results showed that the migration of the A549 and H1299 cells was obviously inhibited by both COL5A2 siRNA and EPHB2 siRNA administration (Fig. 2K, L). The quantitative statistics of the Transwell assay were also similar, and inhibition of COL5A2 and EPHB2 significantly suppressed the migration of LUAD cells (Fig. 2M–P). These data confirmed that both COL5A2 and EPHB2 inhibition also effectively blocked the proliferation and migration of LUAD cells.

### Constructing an angiogenesis-related prognostic model and validating its predictive performance

**Fig. 3:**
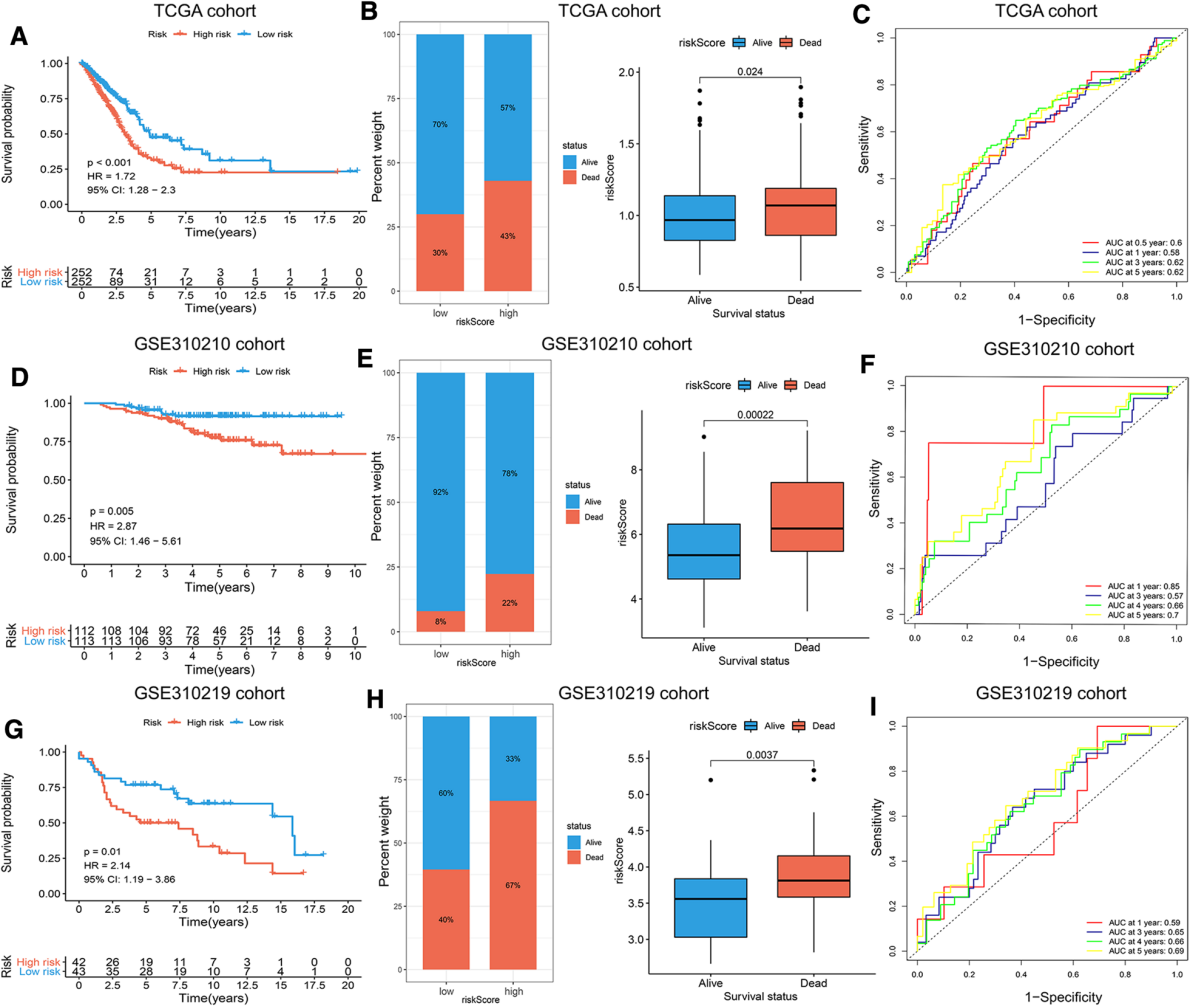
Construction and validation of the angiogenesis-related prognostic model. **A**, **D**, **G** Kaplan–Meier (K–M) survival curves showing the survival status between the high-risk group and low-risk group in the TCGA cohort (**A**), GSE310210 cohort (**D**), and GSE310219 cohort (**G**). **B**, **E**, **H** The bar-plot and box-plot indicated the relationship with the risk score of angiogenesis-related signature and survival status in the TCGA cohort (**B**), GSE310210 cohort (**E**), and GSE310219 cohort (**H**). **C**, **F**, **I** The ROC curve revealed the AUC value of the prognostic model in the TCGA cohort (**C**), GSE310210 cohort (**F**), and GSE310219 cohort (**I**)

The two angiogenesis-related prognostic signatures were established based on multivariate Cox regression analysis and two angiogenic genes (COL5A2 and EPHB2). The prognostic index (PI) was identified as follows: PI = (0.099 * expression level of COL5A2) + (0.182 * expression level of EPHB2). The optimal cutoff value was determined by the median PI and the TCGA-LUAD cohort (including 513 cases with survival time and status data) was divided into high-risk and low-risk groups. The survival curves showed that the OS of the high-risk group was obviously worse than that of the low-risk group (p < 0.001, HR = 1.72, 95% CI 1.28–2.30) (Fig. 3A). To further confirm the prediction ability and good predictive value of these two angiogenic gene prognostic signatures, we used the GSE310210 and GSE310219 cohorts from the GEO database as validation cohorts. The survival curves indicated that the OS in the high-risk group was also significantly worse in the GSE310210 cohort (p = 0.005, HR = 2.87, 95% CI 1.46–5.61) (Fig. 3D) and in the GSE310219 cohort (p = 0.01, HR = 2.14, 95% CI 1.19–3.86) (Fig. 3G). The bar plot and box plot indicated that the survival status was worse in patients with higher risk scores in the TCGA cohort (Fig. 3B), GSE310210 cohort (Fig. 3E) and GSE310219 cohort (Fig. 3H). In addition, the area under the curve (AUC) at 0.5, 1, 3, and 5 years was 0.60, 0.58, 0.62, and 0.62 respectively, in the TCGA cohort (Fig. 3C), and the AUCs of the GSE310210 cohort and GSE310219 cohort reached 0.85, 0.57, 0.66, and 0.7 (Fig. 3F) and 0.59, 0.65, 0.66, and 0.69 (Fig. 3I) for the 1-, 3-, 4- and 5-year survival times, respectively. These results showed that the OS in the high-risk group was worse than that in the low-risk group and that the two angiogenesis-related prognostic models had a high specificity and sensitivity.

### Validating the chemotherapy response of LUAD patients in the high- and low-risk groups

**Fig. 4:**
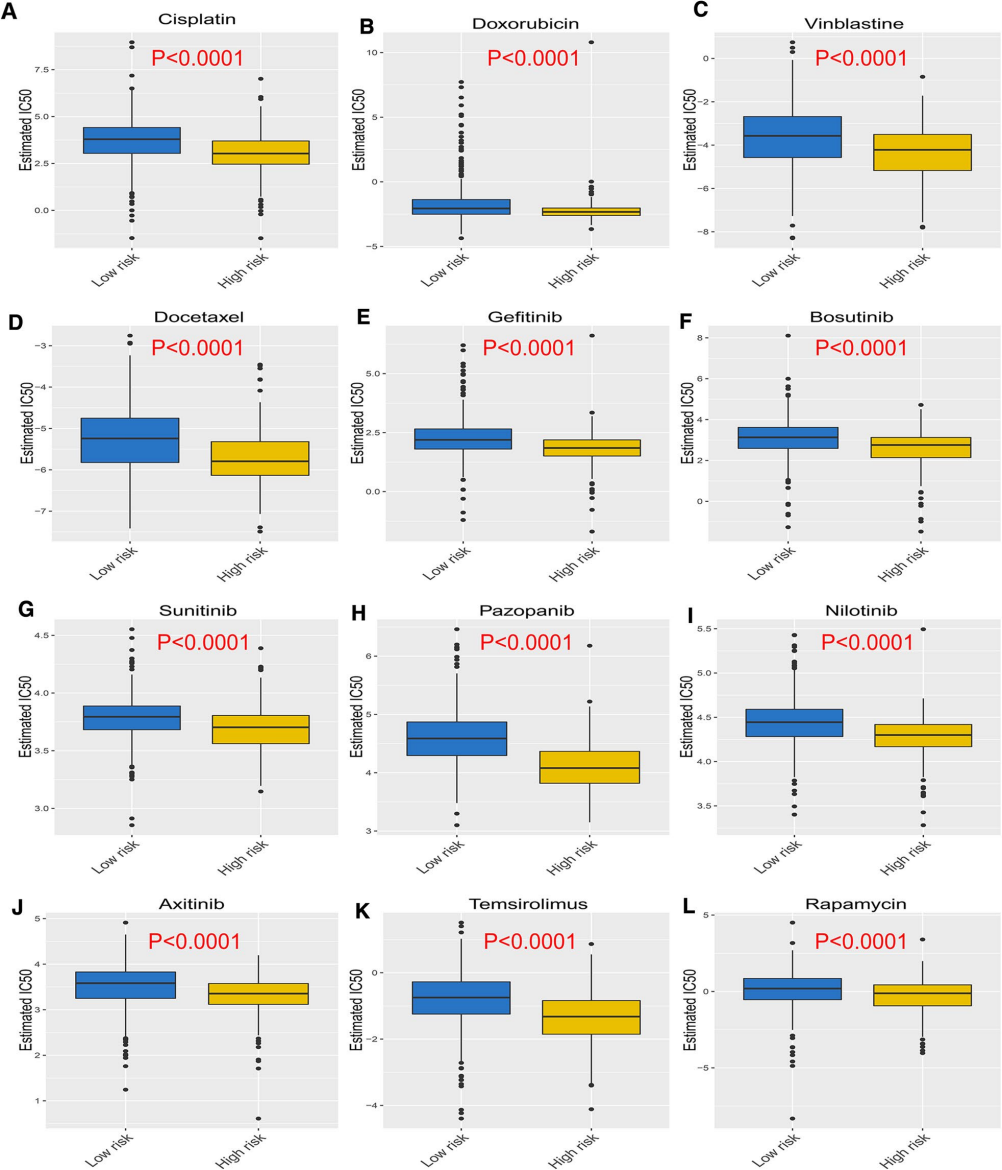
Evaluation of the response sensitivity of chemotherapeutic drugs and molecular targeted drugs in high- and low-risk patients. **A**–**D** Therapeutic response to chemotherapy drugs between high- and low-risk patients. **E**–**L** Therapeutic response of targeted gene drugs of patients with LUAD in high- and low-risk groups

Chemotherapy is the most common treatment therapy for LUAD patients, applied to approximately 90% of cases. The therapeutic response to chemotherapy is an issue deserving of attention. Based on the Genomics of Drug Sensitivity in Cancer (GDSC) website, we obtained the pharmacological effect of 266 traditional chemotherapeutic drugs and molecular-targeted drugs and matched them with the prognostic model of LUAD patients from the TCGA LUAD cohort. We used the half-maximum inhibitory concentration (IC50) as the main criterion for cancer sensitivity to drugs. Figure 4A–D shows the IC50 values of chemotherapeutic drugs, including cisplatin, doxorubicin, vinblastine and docetaxel in the high-risk group of LUAD patients was lower than those in low-risk group (P < 0.0001), revealing that the high-risk group of LUAD patients was more sensitive to the chemotherapeutic drugs. Similarly, the IC50 values of molecular targeted drugs such as gefitinib, bosutinib, and sunitinib in the high-risk group of LUAD patients was much lower comparing to the low-risk group (P < 0.0001) (Fig. 4E–L), indicating that the high-risk group of LUAD patients was more sensitive to the molecular targeted drugs. These results suggest that the high-risk group of LUAD patients may benefit more from these chemotherapeutic drugs and molecular targeted drugs.

### Comparison of the tumor mutation burden of LUAD patients between the high-risk and low-risk groups

**Fig. 5:**
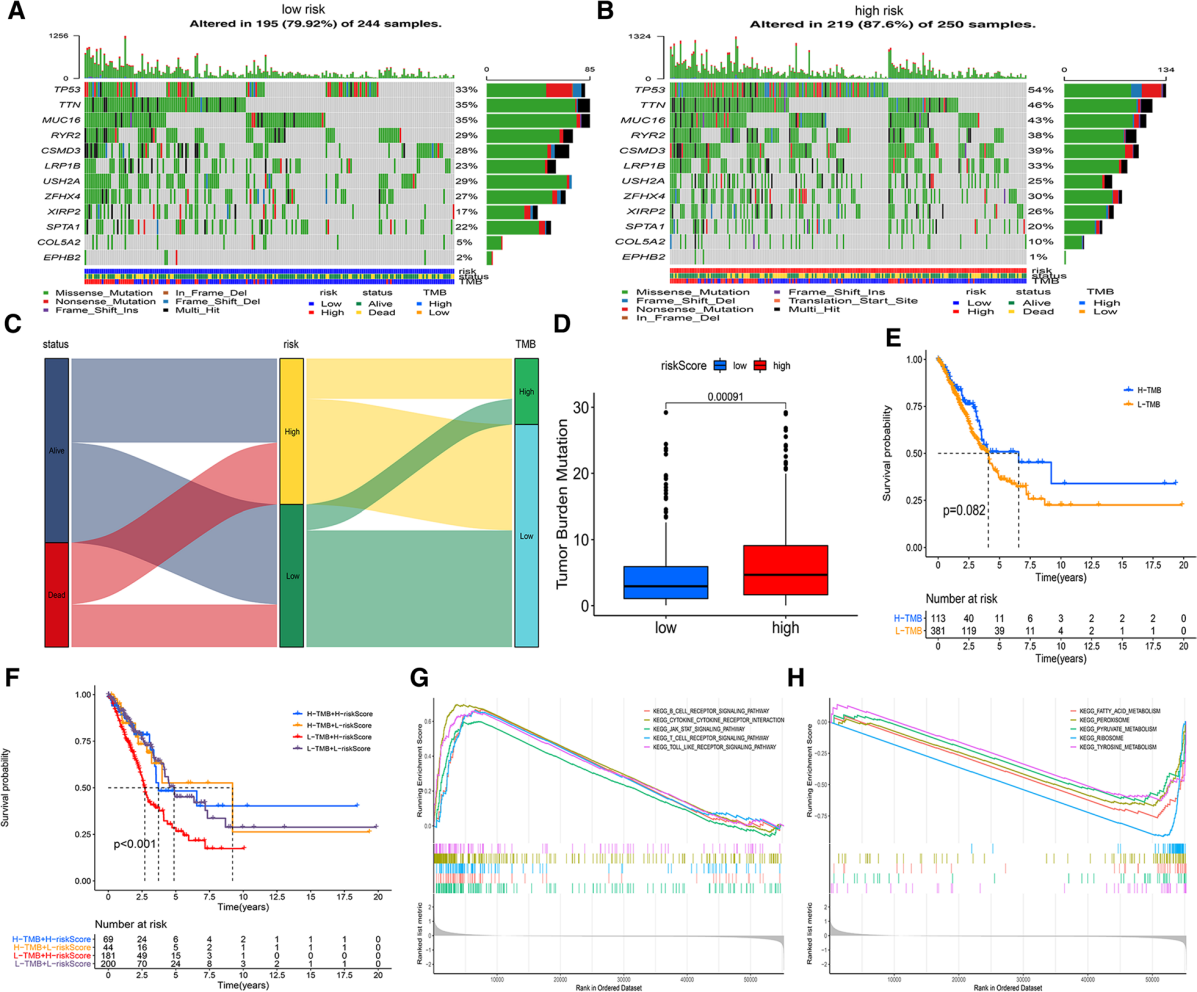
Validation of the correlation with tumor mutation burden and risk score of angiogenesis-related signature in LUAD patients. **A**, **B** The waterfall plot shows the mutation landscape of the top 10 genes and two angiogenic genes in LUAD patients with low-risk scores (**A**) and high-risk scores (**B**). **C**, **D** The Sankey diagram and box plot indicate the correlation with tumor mutation burden and risk score in LUAD patients. **E**, **F** Kaplan–Meier (K-M) survival curves revealed the prognostic value of TMB with or without combination with the risk score in LUAD patients. **G**,** H** GSEA revealed the positive signaling pathway (**G**) and negative signaling pathway (**H**) in high-risk patients

Increasing amounts of evidence has shown that tumor mutation burden (TMB) can be regarded as a biomarker for predicting the response to immunotherapy of LUAD patients [22]. In this study, we analyzed the correlation of TMB and the risk score of the angiogenic gene signature among LUAD patients. Figure 5A, B shows the landscape of the top 10 mutated genes and two angiogenic genes, COL5A2 and EPHB2, from the TCGA-LUAD cohort in the high-risk and low-risk groups. The patients in the high-risk group had higher mutation frequencies of TP53, TTN, MUC16, and COL5A2 than patients in the low-risk group. Figure 5C–D indicates the relationship between TMB and risk score in LUAD patients, and the high-risk group had a higher TMB than the low-risk group. Then, we identified whether TMB was an independent biomarker for LUAD patients. The TCGA-LUAD cohort was divided into two groups, the high TMB group and the low TMB group, by using the “survminer” R package. As a result, TMB was not an independent factor for LUAD patients (Fig. 5E). However, a combination of the risk score and TMB effectively predicted the outcomes of patients with LUAD (Fig. 5F). In addition, GSEA showed the signaling pathways that were positively and negatively correlated in the high-risk group. Compared with the low-risk group, the high-risk group exhibited a positive correlation with immune-related signaling pathways, such as the JAK-STAT signaling pathway, B cell receptor signaling pathway, and T cell receptor signaling pathway, in LUAD patients (Fig. 5G, H).

### Identification of the tumor microenvironment of patients with LUAD in the high- and low-risk groups

**Fig. 6:**
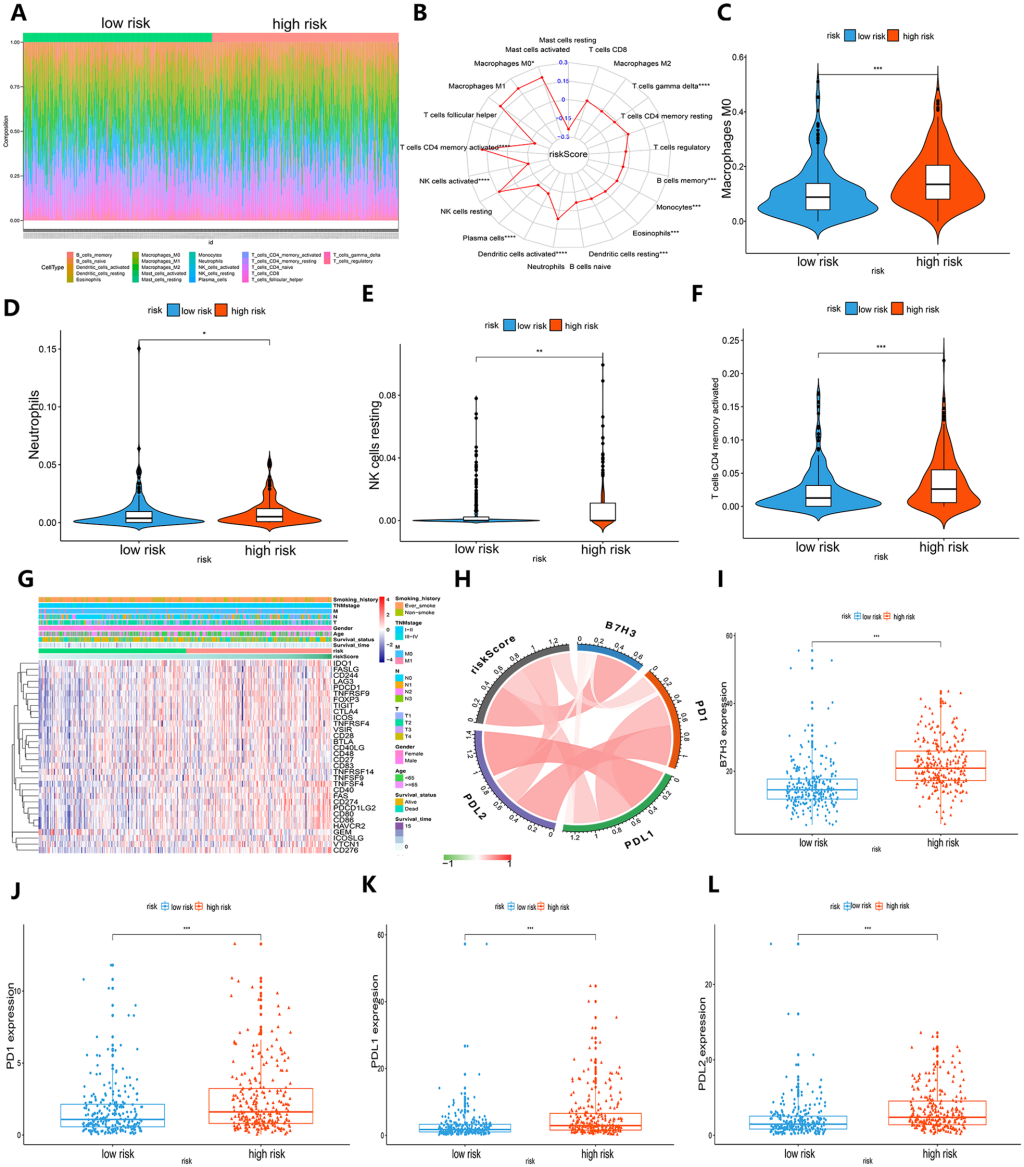
Comparison of the difference in the tumor microenvironment in high- and low-risk patients.** A** Heatmap showing the abundance of immune cell infiltration between high- and low-risk patients. **B** The radar chart shows the correlation with immune cell infiltration and the risk score of the angiogenic gene signature in LUAD patients. **C–F** The box plot indicates the abundance of infiltrating immune cells, including M0 macrophages (**C**), neutrophils (**D**), resting NK cells (**E**), and activated memory CD4 T cells (**F**), between high- and low-risk patients. **G** The heatmap revealed the expression level of immune checkpoints in the high-risk group and low-risk group. **H** The circle chart shows the relationship between the level of immune checkpoints and risk score in LUAD patients. **I–L** The box plot indicates the expression level of immune checkpoints, such as B7H3 (**I**), PD-1 (**J**), PDL-1 (**K**), and PDL-2 (**L**), in high-risk and low-risk patients

To confirm the immunologic features of patients with LUAD in the high-risk and low-risk groups, we used CIBISORT. R package to identify the immune infiltration of 22 immune cell types of patients with LUAD from the TCGA-LUAD cohort. Figure 6A, B shows the relationship of the proportion of infiltration of 22 immune cell types and the angiogenic model prognosis. Compared with the low-risk group, tumor-infiltrating immune cells showed a higher proportion, including M0 macrophages, neutrophils, resting NK cells, and activated memory CD4 T cells (Fig. 6C–F). In addition, the heatmap and circle diagram indicate the close correlation between the immune checkpoints and angiogenic prognostic risk scores (Fig. 6G, H). Compared with the low-risk group, the expression levels of the immune checkpoints PD-1, PDL-1, PDL-2, and B7H3 were obviously higher in the high-risk group (Fig. 6I–L). These results showed that the higher risk patients with LUAD may obtain more clinical benefits from immunotherapy.

### Construction and evaluation of the predictive nomogram in patients with LUAD from TCGA

**Fig. 7:**
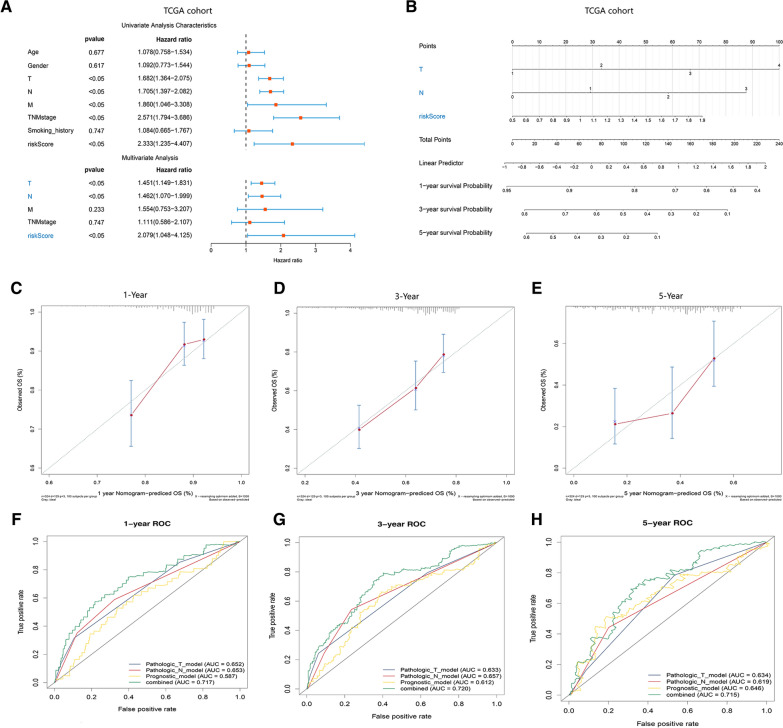
Establishment and evaluation of the predictive nomogram in patients with LUAD. **A** Forest plot indicating independent prognostic clinical factors in LUAD patients. **B** The nomogram of independent prognostic factors was constructed to predict the survival probability at 1 year, 3 years and 5 years of LUAD patients. **C**–**E** The calibration charts revealed the predictive ability of the nomogram for 1 year (**C**), 3 years (**D**) and 5 years (**E**). **F**–**H** The ROC curve exhibited the predictive performance of each independent predictive factor and nomogram for 1 year (**F**), 3 years (**G**) and 5 years (**H**)

To further determine the independent role of the prognostic signature in predicting OS, we used univariate and multivariate PH Cox regression to evaluate the angiogenic prognostic signature and other clinical features (age, sex, T stage, M stage, N stage, TNM stage and smoking history) of patients with LUAD in the TCGA-LUAD cohort. Figure 7A shows that T stage, N stage and the risk score of the angiogenic prognostic signature were independent prognostic factors in predicting OS (P < 0.05). Then, we used independent prognostic factors, including T stage, N stage and risk score, to establish a nomogram to predict the survival probability of LUAD patients at 1, 3 and 5 years (Fig. 7B). The calibration curves of the nomogram were close to the best prediction curve and indicated that the predictive ability was highly consistent at 1, 3 and 5 years for LUAD patients (Fig. 7C–E). We also used ROC curves to validate the predictive value of the nomogram, and the AUC values of the nomogram at 1, 3, and 5 years were 0.717, 0.720 and 0.715, respectively (Fig. 7F–H). These results showed that the risk score of the angiogenic prognostic signature is an independent prognostic factor and that the predictive nomogram of LUAD patients has high specificity and sensitivity.

### Establishment and validation of a diagnostic model based on two angiogenic genes

**Fig. 8:**
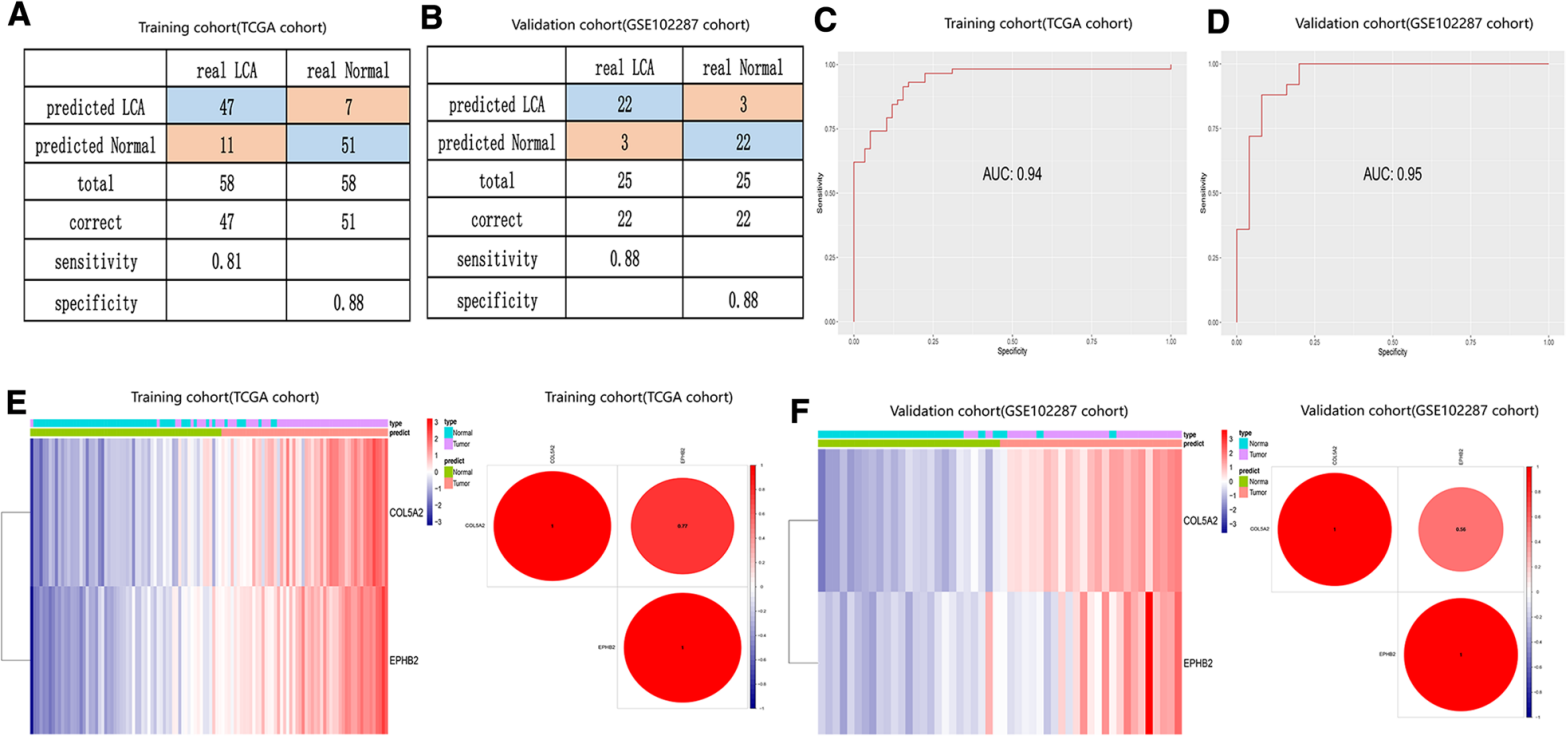
Construction of a diagnostic model for distinguishing LUAD from normal samples. **A**,** B** Confusion matrix chart showing the sensitivity and specificity of the diagnostic model to distinguish LUAD from normal samples in the TCGA-LUAD cohort (**A**) and GSE102287 cohort (**B**). **C**, **D** The ROC curve indicates the predictive value of the diagnostic model in the TCGA-LUAD cohort (**C**) and GSE102287 cohort (**D**). **E**, **F** The heatmap and the correlation analysis show the relationships COL5A2 and EPHB2 in the TCGA-LUAD cohort (**E**) and GSE102287 cohort (**F**)

To confirm the diagnostic ability of these two angiogenic genes in LUAD patients, we used stepwise logistic regression analysis to establish a diagnostic model to distinguish LUAD samples from normal subjects. The diagnostic score was finally identified as follows: logit (P = LUAD) = − 18.049 + (1.117 × COL5A2 expression level) + (1.453 × EPHB2 expression level). The diagnostic model showed that the predicted sensitivity and specificity were 0.81 and 0.88 in the TCGA-LUAD cohort (including 58 normal samples and 58 paired tumor samples) (Fig. 8A) and 0.88 sensitivity and 0.88 specificity in the GSE102287 cohort (containing 25 normal samples and 25 paired tumor samples) (Fig. 8B). The AUCs of the ROC curve from the TCGA-LUAD cohort and GSE102287 cohort were 0.94 and 0.95, respectively (Fig. 8C, D). Unsupervised hierarchical clustering of the two angiogenic genes showed a superior ability to differentiate LUAD from normal samples. The expression levels of these two angiogenic genes indicated a close relationship in the diagnostic model (Fig. 8E, F). These results indicate that the diagnostic model can accurately distinguish LUAD patients from normal subjects with high specificity and sensitivity.

### External and experimental validation to confirm the expression and independent prognostic value of COL5A2 and EPHB2 in LUAD patients

**Fig. 9:**
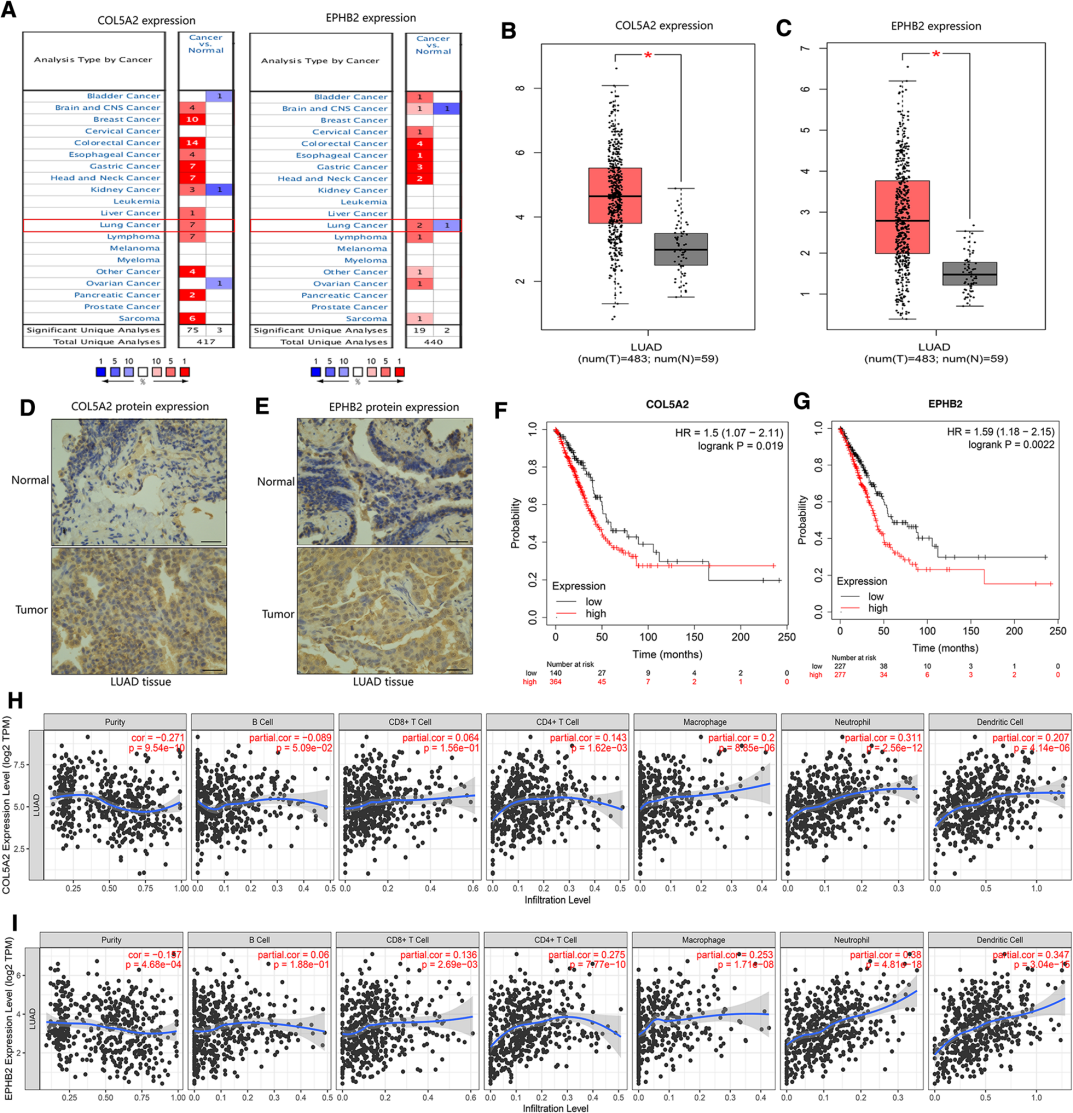
External and experimental validation of COL5A2 and EPHB2 expression levels and independent prognostic value in LUAD patients. **A** Oncomine database showing the transcriptomic aberration of COL5A2 and EPHB2 in pancancer tissues. **B**, **C** The GEPIA database exhibiting the transcriptomic aberration of COL5A2 and EPHB2 in LUAD tissues and nontumor tissues. **D**, **E** IHC assay indicates the protein expression levels of COL5A2 and EPHB2 in LUAD tissues and nontumor tissues. **F**,** G** Kaplan–Meier Plotter database revealed the independent prognostic value of COL5A2 (**F**) and EPHB2 (**G**) in lung cancer patients. **H**, **I** TIMER database showing the correlation between immune cell infiltration and COL5A2 (**H**) and EPHB2 (**I**) in LUAD patients

We confirmed the transcriptomic aberrations of these two angiogenic genes, COL5A2 and EPHB2, in LUAD patients from the Oncomine database. The results showed that the expression levels of COL5A2 and EPHB2 were obviously upregulated in lung cancer tissue compared with normal tissue (Fig. 9A). We also obtained the expression pattern of COL5A2 and EPHB2 from the GEPIA database. Similarly, compared with normal samples, the levels of COL5A2 and EPHB2 were significantly increased in LUAD samples (Fig. 9B, C). To further confirm the expression of COL5A2 and EPHB2 in lung cancer, an immunohistochemistry assay was used to detect the levels of these two angiogenic genes. The results indicated that the expression of COL5A2 and EPHB2 was obviously elevated in lung cancer tissue (Fig. 9D, E). Then, K–M curve analysis was performed to assess the predictive ability of these two angiogenic genes in lung patients from the Kaplan–Meier Plotter database. The results showed that higher levels of COL5A2 and EPHB2 was correlated with worse survival status of lung cancer patients (Fig. 9F, G). By using the Timer database, we also identified the correlation between the expression of these two angiogenic genes and tumor-infiltrating immune cells in patients with LUAD (Fig. 9H, I). These results showed that the levels of COL5A2 and EPHB2 were upregulated in lung cancer tissue and were regarded as independent prognostic factors in lung cancer patients.

## Discussion

LUAD is the most prevalent subtype of lung cancer with high mortality and morbidity worldwide. The survival rate of LUAD patients is very poor; after diagnosis, less than 50% of cases survive one year, and the five-year survival rate is just 18% [23]. The most common cause is that LUAD is diagnosed at advanced stages involving extensive metastatic tumors [24]. In this study, we identified early diagnostic and prognostic biomarkers to predict clinical outcomes based on angiogenesis-related genes in LUAD patients. We found that patients with LUAD in the high-risk group had a worse prognosis than patients in the low-risk group in both the training cohort and validation cohort. Based on the risk score of the angiogenesis gene signature and other interdependent clinical features, we established a nomogram model with great prediction performance. In addition, the angiogenesis gene signature can accurately distinguish LUAD samples from nontumor samples with superior specificity and sensitivity. These findings suggest that these angiogenesis gene signatures could serve as effective theranostic and prognostic biomarkers with high predictive ability in LUAD patients.

Collagen type V alpha 2 chain (COL5A2) has previously been reported to be involved in various pathological processes in tumorigenesis and cancer progression, such as cancer cell survival, migration, immune microenvironment, angiogenesis and blood vessel development [25, 26]. The expression level of COL5A2 can also effectively predict patient outcomes in multiple malignant tumors, including gastric cancer, bladder cancer and tongue squamous cell carcinoma [27–29]. EPHB2 is a member of the erythropoietin-producing hepatocellular (Eph) receptor family, which plays a critical role in various physiological and pathological functions, such as cell adhesion and migration, stemness and angiogenesis [30–32]. A large number of studies have demonstrated the independent prognostic significance of EPHB2 expression in multiple malignancies, such as colorectal cancer, breast cancer, gastric cancer and rectal cancer [33]. In this study, we identified these two angiogenesis genes (COL5A2 and EPHB2) to establish diagnostic and prognostic models with great predictive performance in LUAD patients. Furthermore, we also knocked down the expression levels of COL5A2 and EPHB2 in LUAD cells, including A549 and H1299 cells. These cell experiments indicated that inhibition of COL5A2 and EPHB2 obviously inhibited the proliferation and migration of LUAD cells. These results showed that the angiogenesis gene signature has great diagnostic performance and prognostic value in LUAD patients and that COL5A2 and EPHB2 may be regarded as potential therapeutic targets for patients with LUAD.

Immunotherapies involving immune checkpoint inhibitors (ICIs) have great clinical efficacy in some solid tumors, including LUAD, and have revolutionized the management of cancer [34]. Increasing evidence suggests that TMB is closely associated with tumor immunogenicity and has been proven to be an indicator of ICI therapy in non-small cell lung cancer [22]. In this study, we found that patients with LUAD in the high-risk group showed a higher tumor mutation burden and higher mutation frequencies of TP53, TTN, MUC16, and COL5A2 than patients in the low-risk group. Furthermore, even though TMB is not a prognostic clinical feature, combining our risk score and TMB can effectively predict the outcome of LUAD patients. In addition, immune checkpoint expression and tumor-infiltrating lymphocytes also serve as useful predictors and effectors of immune checkpoint inhibitors in LUAD patients [35]. In the present study, we found that LUAD patients in the high-risk group revealed higher infiltration of tumor-infiltrating immune cells, such as M0 macrophages, neutrophils, resting NK cells, and activated memory CD4 T cells, than patients in the low-risk group. Additionally, when measuring the expression level of immune checkpoints, higher expression of PD-1, PDL-1, PDL-2, and B7H3 was revealed in LUAD patients in the high-risk group. In addition, the patients in the high-risk group exhibited close associations with immune-related signaling pathways, such as the B cell receptor signaling pathway and T cell receptor signaling pathway. Chemotherapy and targeted gene therapies have been proven to have great benefit in prolonging the survival of patients with LUAD [36, 37]. Therefore, it is important to predict the therapeutic response to chemotherapy drugs and molecular targeted antitumor drugs among LUAD patients. In this study, we found that patients in the high-risk group were more sensitive to some kinds of chemotherapy drugs and molecular targeted drugs, such as cisplatin, doxorubicin, gefitinib, bosutinib and sunitinib. These results suggest that the angiogenesis gene signature is closely associated with immunotherapy and chemotherapy and can be used to select individual treatment strategies for LUAD patients.

In summary, we identified two angiogenesis gene (COL5A2 and EPHB2) signatures and constructed diagnostic and prognostic models with great predictive performance. Additionally, the risk score of the angiogenesis gene signature was closely related to immune cell infiltration, immune checkpoint expression and the therapeutic response to chemotherapy in LUAD patients. Moreover, knocking down the expression levels of COL5A2 and EPHB2 significantly suppressed the proliferation and migration of LUAD cells. Above all, these findings suggested that the angiogenesis gene signature could serve as an effective biomarker correlated with clinical relevance, immune infiltration and cancer progression of LUAD patients.

However, there are some limitations in our current research. First, the RNA sequence data and clinical information of our search were obtained from the TCGA and GEO databases. Second, a feasible cohort to validate the role of the angiogenesis gene signature in the therapeutic response to immunotherapy and chemotherapy among LUAD patients is lacking. Third, the number of LUAD samples was too low to assess the predictive ability of the angiogenesis gene signature model. Fourth, no animal experiments were used to explore the oncogenic effect of COL5A2 and EPHB2 in LUAD patients. Overall, we need more samples and more experiments to further explore and validate our findings.

## Supplementary Information

**Additional file 1:Figure S1.** The flowchart of integrative analysis and experiments to explore angiogenesis regulators correlated with poor prognosis, immune infiltration and cancer progression in lung adenocarcinoma.

**Additional file 2:Table S1.** Angiogenesis related genes.

**Additional file 3:Table S2.** Univariate and multivariate Cox regression analyses of the prognostic genes to OS in LUAD patients.

## Data Availability

The data and material used to support the findings of this study are available from the corresponding author upon request.
