# Supplementary figures and images for: Integrative analysis and experiments to explore angiogenesis regulators correlated with poor prognosis, immune infiltration and cancer progression in lung adenocarcinoma
Source: J Transl Med. 2021 Aug 21;19:361. doi: 10.1186/s12967-021-03031-w (PMC8380343; doi:10.1186/s12967-021-03031-w)

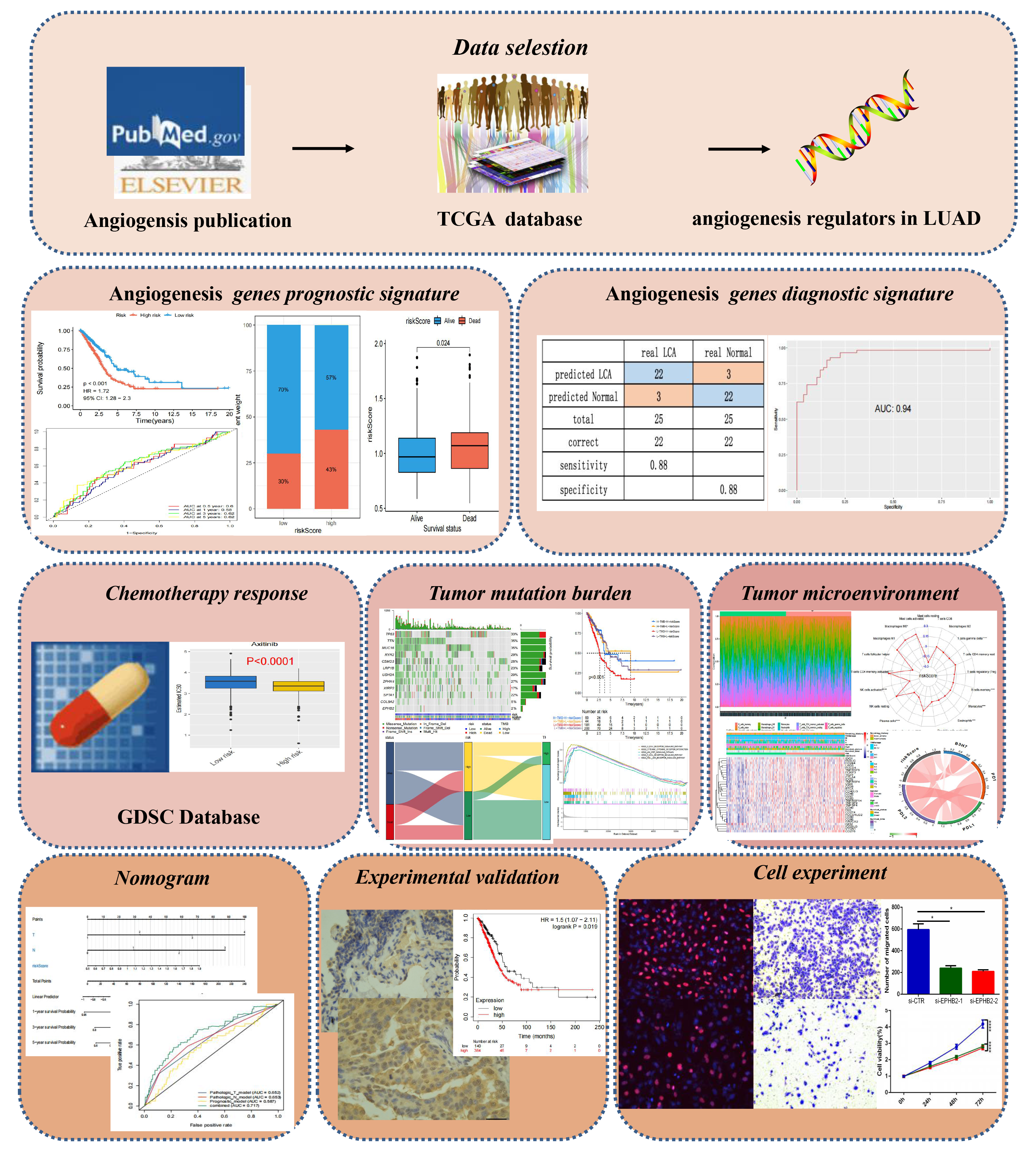

Supplement: Supplementary file 1 — Additional file 1:Figure S1. The flowchart of integrative analysis and experiments to explore angiogenesis regulators correlated with poor prognosis, immune infiltration and cancer progression in lung adenocarcinoma. [file 12967_2021_3031_MOESM1_ESM.tif]
